# Mice deficient in N-acetylgalactosamine 4-sulfate 6-O-sulfotransferase exhibit enhanced liver fibrosis and delayed recovery from fibrosis in carbon tetrachloride-treated mice

**DOI:** 10.1016/j.heliyon.2016.e00138

**Published:** 2016-08-08

**Authors:** Hiroko Habuchi, Takahiro Ushida, Osami Habuchi

**Affiliations:** a Advanced Medical Research Center, Aichi Medical University, 1-1 Yazakokarimata, Nagakute, Aichi 480-1195, Japan; b Multidisciplinary Pain Center, Aichi Medical University, 1-1 Yazakokarimata, Nagakute, Aichi 480-1195, Japan

**Keywords:** Cell biology, Biochemistry

## Abstract

**Background:**

Chondroitin/dermatan sulfate (CS/DS) rich in *N*-acetylgalactosamine 4,6-bissulfate (GalNAc(4,6SO_4_)) residues is present as decorin and/or biglycan in mouse liver, and GalNAc(4,6SO_4_) residues disappeared completely in N-acetylgalactosamine 4-sulfate 6-*O*-sulfotransferase (GalNAc4S-6ST) knockout (KO) mice. The aim of this study was to investigate whether CS/DS rich in GalNAc(4,6SO_4_) residues participate in the progression or resolution of liver fibrosis.

**Methods:**

Wild type (WT) and GalNAc4S-6ST KO mice were treated with CCl_4_ for 5 weeks. After discontinuation of CCl_4_ administration, histochemical and biochemical changes and expression of genes related to matrix components were compared between WT and GalNAc4S-6ST KO mice.

**Results and conclusion:**

On 2 days after cessation of CCl_4_ administration, higher fibrosis was observed in KO mice than in WT mice by Sirius Red staining. Serum alanine aminotransferase activity was higher in KO mice than in WT mice. Hydroxyproline contents and Sirius Red staining showed that repair of liver fibrosis in the recovery stages appeared to be delayed in KO mice. Expression of mRNA of matrix metalloproteinase (MMP)-2, MMP-13 and versican peaked at 2 days after cessation of CCl_4_ administration and was higher in KO mice than in WT mice. Expression of MMP-9 in the recovery stage was lower in KO mice than in WT mice. Our findings demonstrate that defect in GalNAc4S-6ST, which resulted in disappearance of CS/DS containing GalNAc(4,6SO_4_), appear to contribute to progression of liver fibrosis, delayed recovery from fibrosis, and various changes in the expression of proteoglycans and MMPs in carbon tetrachloride–treated mice.

## 1 Introduction

N-acetylgalactosamine 4-sulfate 6-O-sulfotransferase (GalNAc4S-6ST) transfers sulfate to position 6 of GalNAc(4SO_4_) residues of chondroitin sulfate (CS)/dermatan sulfate (DS) to form GalNAc(4,6SO_4_) residues. GalNAc4S-6ST and CS/DS containing GalNAc(4,6SO_4_) residues (CS/DS-E) have been implicated in various biological functions. CS/DS-E was reported to involve in the regulation of neurite outgrowth [1, 2, 3, 4]. CS/DS-E was found to affect the growth and metastatic property of cancer cells [5, 6, 7]. CS/DS-E enhanced differentiation of osteoblast [8, 9], whereas CS/DS-E inhibited differentiation of osteoclast [10]. Downregulation of GalNAc4S-6ST improved left ventricular function and ameliorated the progression of cardiac remodeling in chronic heart failure after experimental autoimmune myocarditis [11]. CS/DS-E enhanced chondrogenic differentiation of ATDC5 cells [12].

We have found previously that highly sulfated CS/DSs in which IdoA-GalNAc(4,6SO_4_) units amount to nearly 40% of the repeating disaccharides are present in the mouse liver as GAG chains of decorin and/or biglycan, and that IdoA-GalNAc(4,6SO_4_) units in CS/DS disappeared completely in GalNAc4S-6ST knockout (KO) mice [13]. Presence of CS/DS-E in the mammalian liver had been reported previously [14, 15]. Decorin and decorin mRNA were reported to increase in the liver fibrosis [16, 17, 18]. In decorin KO mice, liver fibrosis induced by thioacetamide was enhanced and the tissue repair delayed [19]. It was reported that the acute liver damage after CCl_4_ administration was more severe in EXTL2 KO mice in which glycosaminoglycans were overproduced than in wild type mice [20]. In liver cirrhosis of Long–Evans Cinnamon Rats, it was reported that the remarkable increase in the content of oversulfated CS/DS and the decrease in the degree of oversulfation of the CS/DS were observed in the fibrous lesion compared with the nonfibrous lesion [21]. These observations suggest that the highly sulfated CS/DS with GalNAc(4,6SO_4_) residues attached to decorin might contribute to regulation of fibrosis in the liver. In this report, we compared liver fibrosis induced by carbon tetrachloride between GalNAc4S-6ST KO mice and wild type mice, and found that enhanced fibrosis and delayed recovery were observed in GalNAc4S-6ST KO mice.

## 2 Materials and methods

### 2.1 Materials

Heparitinase I (*Flavobacterium heparinum*, EC 4.2.2.8), heparitinase II (*F. heparinum*, no number assigned), heparinase (*F. heparinum*, EC 4.2.2.7), chondroitinase ABC, chondroitinase ACII, unsaturated disaccharides kit for HS (heparan sulfate) or CS were purchased from Seikagaku Corp (Tokyo, Japan). Pronase and collagenase D were from Roche Applied Science (Germany). Deoxyribonuclease I was from Sigma-Aldrich Japan (Tokyo, Japan). Anti-type I collagen antibody was from Cosmo Bio Corp (Tokyo, Japan). Anti-decorin monoclonal antibody (Anti dermatan sulfate proteoglycan mAb, clone 6-B-6) and anti-versican monoclonal antibody (Anti large proteoglycan mAb clone 2-B-1) were from Seikagaku Corp (Tokyo, Japan). AlexaFluor546 conjugated anti-rabbit IgG antibody was from Thermo Fisher Scientific (Yokohama, Japan). Histofine (mouse staining kit) for mouse monoclonal antibody to mouse tissue was from Nichirei (Tokyo, Japan). Envision kit/HRP (DAB) was from DAKO Japan (Tokyo, Japan). Recombinant human TGF-β1 and recombinant murine TNF-α were purchased from PeproTeck (NJ, USA)

### 2.2 Animals and administration of CCl_4_

GalNAc4S-6ST-knockout mice were generated as described previously [13]. Mice used in the CCl_4_-induced fibrosis study had been back-crossed to BALB/c mice for more than 10 generations. Mice used for preparation of hepatic stellate cell (HSC) had been back-crossed to C57BL/6 N mice for more than 10 generations. Mice were maintained in 12-hour light/12-hour dark cycles with free access to food and water in a pathogen-free room at the Laboratory Animal Research Center, Aichi Medical University. Mice (7-to-8-week-old females) were injected intraperitoneally with carbon tetrachloride (CCl_4_), which was diluted 2:5 in mineral oil (Sigma) at a dose of 2.0 ml/kg (as CCl_4_) of body weight twice a week for a total of 9 injections. Control mice were injected with mineral oil alone. Number of mice used for analysis was indicated under the legend of each figure. Mice were killed 2 days, 3 weeks, 6 weeks or 9 weeks after the last CCl_4_ injection, and blood and liver samples were obtained. The liver was either fixed with 10% formaldehyde neutral buffer solution (Nakalai Tesque, Kyoto, Japan) for histological examination or used for extraction of RNA, analysis of glycosaminoglycans and measurement of the content of hydroxyproline. All animal procedures were approved by Animal Research Committee of Aichi Medical University.

### 2.3 Determination of hydroxyproline

Hydroxyproline was determined by the method of Kivirikko et al. [22] as modified by Nagatani et al. [23] after hydrolysis with 6 M HCl at 110 °C for 24 h. In this modified method, reaction with chloramine T was carried out at 0 °C for 2 h.

### 2.4 Assay of alanine aminotransferase (ALT) activity

ALT activity was measured by using an ALT activity assay kit (BioVision) according to the manufacturer’s protocol. To the reaction mixture, 3 μl of the serum was added, and fluorescence (Ex/Em = 535 nm/587 nm) was determined at 20 min and 50 min.

### 2.5 Isolation of total RNA and determination of expression levels of mRNAs by quantitative reverse-transcription polymerase chain reaction (qRT-PCR)

Total RNA was isolated from mouse livers using Trizol (Invitrogen) or isolated from the cultured HSCs using NucleoSpin XS (Macherey-Nagel). A reverse-transcription reaction was performed using the High-capacity cDNA Archive kit (Applied Biosystems, Foster City, CA). Quantitative RT-PCR was performed using commercial specific primer pairs (for decorin, biglycan, versican, collagen (COL)1a1, metalloproteinase (MMP)-2, MMP-9, MMP-13, tissue inhibitor of metalloproteinase (TIMP)-1 and TIMP-2) and SYBR premix Ex TaqII (Takara, Shiga, Japan). Normalization of each transcript was performed using β-actin specific primer pairs (Takara, Shiga, Japan). The PCR products were analyzed in real-time using ABI Prism 7000 system. Two independent experiments were performed in triplicate to obtain the values.

### 2.6 Histological analysis and immunohistochemistry of liver

Tissues were fixed with 10% formaldehyde and embedded in paraffin according to standard histological procedures and cut to 4 μm of sections. Collagen fiber was stained with Sirius Red following nuclear-staining with hematoxylin. Images were captured using a KEYEENCE BZ-9000 microscope. The area stained with Sirius Red was measured using NIH ImageJ64. Briefly, each color picture was split into Red, Green and Blue pictures. Using Green pictures, ratios of areas above a defined threshold to total area were determined. For collagen type I immunostaining, the sections were deparaffinized and then digested with 200 μl per section of chondroitinase ABC (10 mU/ml) at room temperature for 30 min. After blocking with 1% BSA, the sections were incubated with anti-collagen type I antibody as the first antibody at 4 °C overnight. After washing, sections were stained with AlexaFluor546-conjugated anti-rabbit IgG antibody. The nuclei were stained with DAPI. The immunohistochemistry of decorin and versican was carried out according to the following steps: digestion with chondroitinase ABC, incubation with anti-decorin antibody or anti-versican antibody, incubation with peroxidase-conjugated anti-mouse IgG (mouse stain kit), and reaction with DAB.

### 2.7 Isolation of glycosaminoglycans from liver and its structural analysis

The liver (about 400 mg) was homogenized in acetone and the acetone-insoluble materials were dried. The dried materials were suspended in 2 ml 0.5 M NaOH and stirred for 16 h at room temperature. After neutralization with 3 M acetic acid, Actinase (0.5 mg/ml) and Tris-HCl, pH 8.0 (final 20 mM) were added and incubated at 37 °C for 24 h. After heating at 100 °C for 5 min, the samples were centrifuged at 10000 x g for 10 min. To the resulting supernatant fractions were added 3 volumes of ethanol containing 1.3% potassium acetate, and glycosaminoglycans were precipitated by centrifugation at 10000 x g for 10 min. The precipitates were dissolved in 1 ml 20 mM Tris-HCl, pH 7.4 containing 20 mM MgCl_2_, DNase I (0.1 mg/ml) and RNase A (0.1 mg/ml), and incubated for 2 h at 37 °C. After heating at 100 °C for 2 min, the samples were centrifuged at 10000 x g for 10 min. From the resulting supernatant fractions, glycosaminoglycans were recovered by ethanol precipitation as above, dissolved in 1 ml 20 mM Tris-HCl, pH 7.2 containing 0.2 M NaCl and loaded onto a DEAE-Sephacel column (bed volume 0.8 ml) equilibrated with the same buffer. The column was washed with 10 column volumes of 20 mM Tris-HCl, pH 7.2 containing 0.2 M NaCl and then eluted with 3 column volumes of Tris-HCl, pH 7.2 containing 2 M NaCl. From the eluates, glycosaminoglycans were recovered by ethanol precipitation as above. The purified glycosaminoglycans were dissolved in 100 μl 50 mM sodium acetate buffer, pH 5.0 containing 1.0 TRU Streptomyces hyaluronidase, and digested for 2 h at 37 °C. After digestion, 2 volumes of ethanol containing 1.3% potassium acetate were added and the mixtures were left at −80 °C for 30 min. Glycosaminoglycans were precipitated by centrifugation at 10000 x g for 30 min at 4 °C. For disaccharide composition analysis of CS/DS, the purified glycosaminoglycans were digested with a mixture of 30 munits chondroitinase ABC and 30 munits chondroitinase ACII in 25 μl of 50 mM Tris-acetate buffer, pH 7.5, 0.1 mg/ml of bovine serum albumin for 4 h at 37 °C. For disaccharide composition analysis of HS/heparin, the purified glycosaminoglycans were digested with a mixture of 0.2 mU of heparitinase I, 0.1 mU of heparitinase II and 0.2 mU of heparinase in 50 μl of 50 mM Tris-HCl buffer, pH7.2, 1 mM CaCl_2_ and 5 μg bovine serum albumin for 2 h at 37 °C. After filtration of the digests with Ultrafree-MC (5 kDa molecular weight cutoff filter: Millipore Corp.), the unsaturated disaccharide products in the filtrates were analyzed by reverse-phase ion-pair chromatography using Senshu Pak column Docosil with a fluorescence detector according to Toyoda’s method [24] with slightly modified elution conditions.

### 2.8 Isolation of hepatic stellate cells from wild type- and KO-mouse liver

Hepatic stellate cells from the mouse liver were prepared according to the method of Machmeyer et al. [25] with a slight modification. Briefly, livers were perfused successively with PBS containing 0.09% glucose, 0.5 mM EGTA and 0.0006% phenol red for 5 min, DMEM/F12 containing 0.4 mg/ml of pronase E for 5 min and DMEM/F12 containing 0.4 mg/ml of collagenase D for 8 min at a flow rate of 5 ml/min. Dissected livers were placed in 10 ml of DMEM, cut into small pieces, and filtrated through 70-μm cell strainer. The cell strainer was washed once with 5 ml DMEM containing DNase I. The pooled filtrate was centrifuged at 50 x g for 2 min. After the supernatant was moved to a new tube, pellet was suspended in 5 ml of DMEM and centrifuged again. Both the supernatants were pooled and centrifuged at 700 x g for 10 min. The cell pellet was suspended in 10 ml 9% (w/v) HistoDenz (Sigma-Aldrich). After loading 1.5 ml of DMEM on the top of the suspension, the cell suspension was centrifuged at 1400 x g for 17 min. HSCs were recovered from the interface between HistoDenz and top layer, and plated in DMEM containing 10% FBS. Two hours after plating, the culture medium containing unattached cells was replaced with fresh medium. More than 90% of the cells thus obtained showed starlike morphology with a lot of lipid droplets, which are characteristic of HSCs.

### 2.9 Effects of TGF-β1 and TNF-α on the expression of mRNAs in the cultured Hepatic stellate cells (HSCs) obtained from wild type- and KO-mouse liver

HSCs cultured for 6 days were seeded in 24-well plates at 1 × 10^4^ cells/well. Two days after seeding, the wells were washed with DMEM once and DMEM containing 0.4% FBS (medium A) was added to each well. After incubation for 24 h, the medium was replaced with fresh medium A containing TGF-β1 (10 ng/ml) or TNF-α (10 ng/ml) and incubation was continued for further 8 h. The cells were harvested and total RNA was prepared. Expression of the mRNAs of COL1a1 and MMP-13 were analyzed.

### 2.10 Gelatin-substrate zymography

Frozen livers were homogenized by a polytron homogenizer in 10-fold extraction buffer (50 mM Tris-HCl, pH 7.5, 0.15 M NaCl, 0.02%Triton X-100) for 30 sec, and the homogenates were then shaken for 30 min at 4 °C. The homogenates were centrifuged at 12,000 rpm for 15 min. The protein concentration of the supernatants was determined by DC Protein Assay kit (Bio-Rad). Aliquots (30 μg of protein) of each sample were separated in 7.5% SDS-polyacrylamide gel containing 1 mg/ml gelatin. After electrophoresis, the gels were washed with 2.5% Triton X-100 for 30 min, and then shaken in a development solution (50 mM Tris-HCl pH 7.5, 5 mM CaCl_2_, 0.2 M MgCl_2_) at 70 rpm for 30 min. The gels were incubated in the fresh development solution at 37 °C for 6 h. After incubation, the gels were stained with 0.5% (w/v) CBBR-250 for 1 h, and then destained with a solution (50% methanol, 10% acetic acid, 40% H_2_O). The gelatinase activity was determined by measurement of the intensity of the transparent bands using NIH Image software.

### 2.11 Statistical analysis

Comparisons between groups were assessed by Student’s *t* test. Values of *p* < 0.05 were considered to be statistically significant.

## 3 Results

### 3.1 Deficient in GalNAc4S-6ST results in advanced fibrosis and delayed recovery from fibrosis

**Fig. 1:**
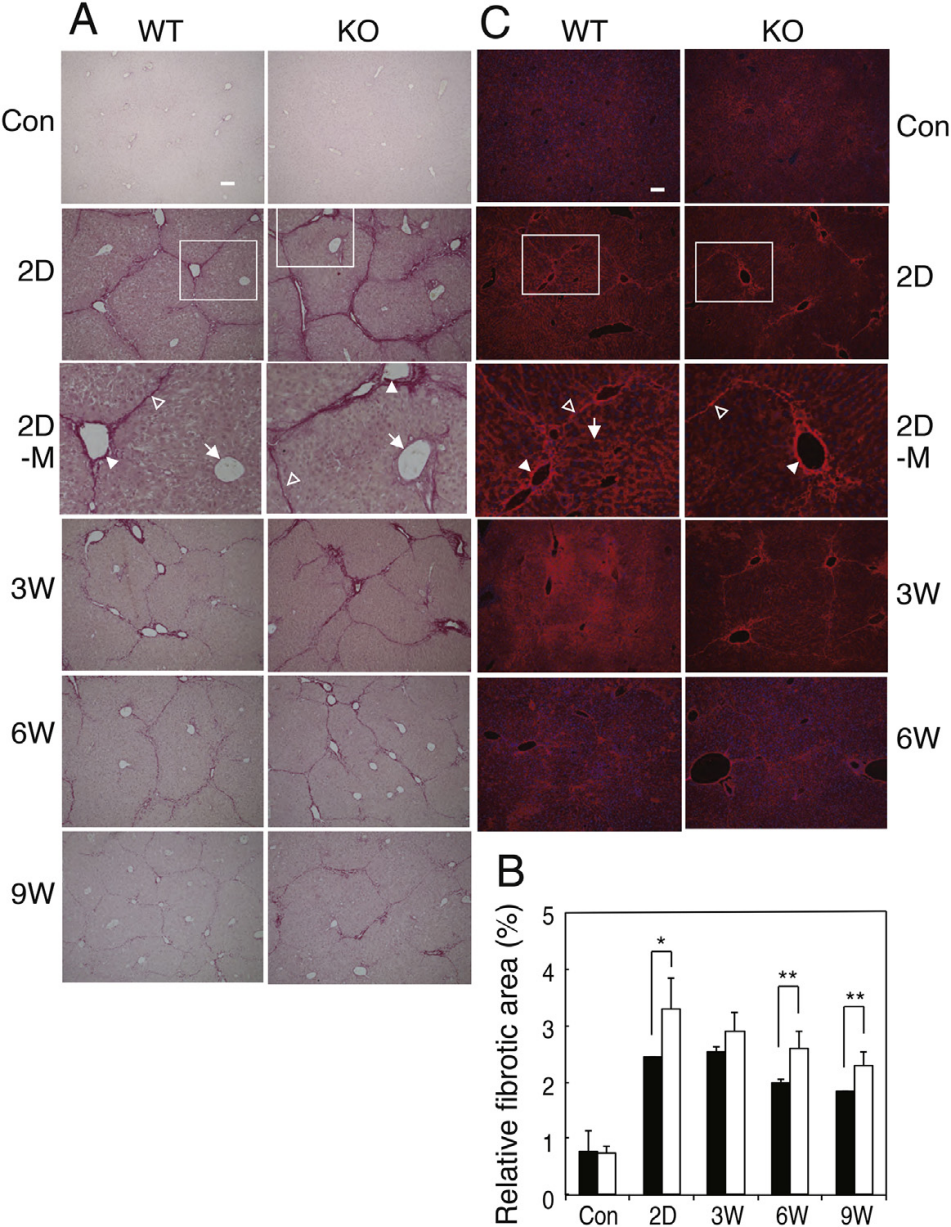
Sirius Red staining and detection of COL1 by immunostaining of the livers from wild type mice (WT) and GalNAc4S-6ST KO mice (KO). (A) Sirius Red staining. Magnified pictures of regions surrounded by rectangles in 2D are shown in 2D-M. Portal tracts, boundaries of hepatic lobule and central veins are indicated by filled arrowheads, open arrowheads and arrows, respectively in 2D-M. Bar: 100 μm. (B) Relative fibrosis determined from the Sirius Red staining. The histograms show the ratios (percentage) of areas above a defined threshold to total areas from wild type (filled bar) and KO mice (open bar). Values indicate averages of data obtained from three mouse livers. Five different pictures were measured per each mouse. SDs of data are indicated above each bar. *p < 0.01, **p < 0.05. (C) Immunostaining of COL1 by anti-COL1 antibody. Bar: 100 μm.

**Fig. 2:**
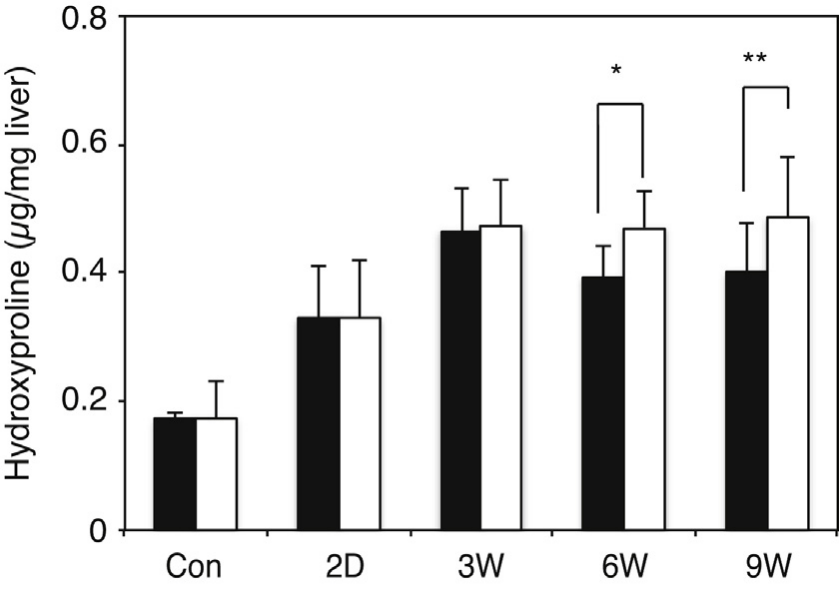
Determination of hydroxyproline in the livers obtained from wild type mice and GalNAc4S-6ST KO mice. The histograms show the hydroxyproline contents of the livers from wild type (filled bar) and KO mice (open bar). Number of mice used for experiments was 3, 5, 7, 8, and 9 for control, 2 days, 3 weeks, 6 weeks and 9 weeks, respectively. Values indicate averages of data obtained from these mice, and SDs of data are indicated above each bar. *p < 0.01, **p < 0.03.

Mice were injected intraperitoneally with carbon tetrachloride (CCl_4_) twice a week for a total of 9 injections as described under “Experimental Procedures”. Control mice were injected with mineral oil alone. Sirius Red staining showed more advanced liver fibrosis and delayed recovery in GalNAc4S-6ST KO mice than in wild type mice (Fig. 1A). Before injection of CCl_4_, Sirius Red hardly stained livers from both wild type and GalNAc4S-6ST KO mice. On 2 days after the last CCl_4_ injection, Sirius Red clearly stained portal tracts and boundary of hepatic lobule, but hardly stained peripheral regions of the central veins. On 3 weeks and thereafter, the stain faded gradually. On 2 days, 6 weeks and 9 weeks after the last CCl_4_ injection, relative fibrosis obtained from Sirius Red staining was significantly higher in GalNAc4S-6ST KO mice than in wild type mice (Fig. 1B). Hydoxyproline contents in the liver of wild type and GalNAc4S-6ST KO mice were determined 2 days, 3 weeks, 6 weeks and 9 weeks after the last CCl_4_ injection (Fig. 2). The amounts of hydroxyproline increased up to 3 weeks in wild type mice and decreased thereafter, whereas they reached a plateau on 3 weeks in GalNAc4S-6ST KO mice. As a result, the hydroxyproline contents were significantly higher in GalNAc4S-6ST KO mice than in wild type mice on 6 weeks and 9 weeks after the last CCl_4_ injection. These observations also suggest that defect in CS/DS-E may result in delayed recovery from fibrosis.

### 3.2 Liver damage induced by CCl_4_ administration was more severe in KO mice than in WT mice

**Fig. 3:**
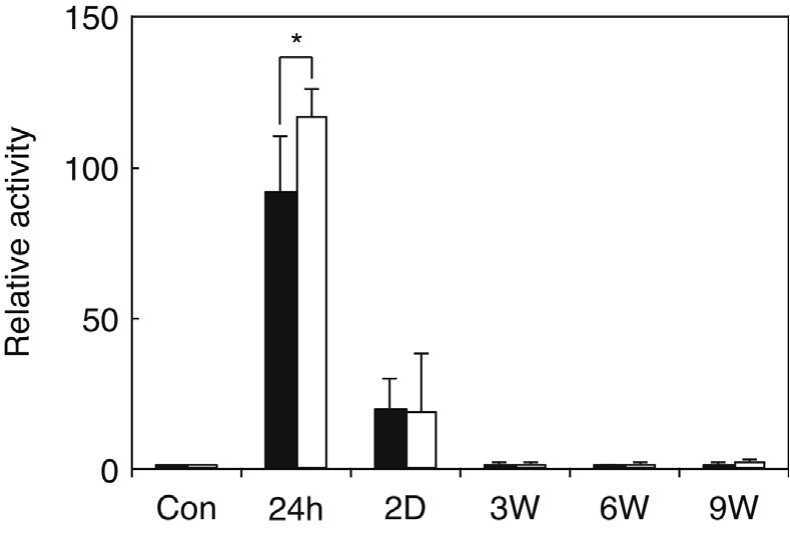
Determination of activities of alanine aminotransferase in the serum obtained from wild type mice and GalNAc4S-6ST KO mice. Mice were treated as described under “Materials and methods” and serum samples of control and CCl_4_-treated mice were obtained. The histograms show the activity of wild type (filled bar) and KO mice (open bar) (*p < 0.03). Values indicate averages of 3 mice, and SDs of data are indicated above each bar.

It is well known that activity of serum alanine aminotransferase (ALT) increases on liver injury. We determined whether the activity of the enzyme induced by the administration of CCl_4_ might be affected by deficient in GalNAc4S-6ST (Fig. 3). At 24 h after the last CCl_4_ injection, ALT activities were elevated markedly, and on three week and thereafter these activities were decreased to the control level; the activity at 24 h was significantly higher in GalNAc4S-6ST KO mice than in wild type mice (Fig. 3B). These results suggest that defect in GalNAc4S-6ST, which resulted in the disappearance of CS/DS-E, may make the liver more susceptible to the harmful effects of CCl_4_.

### 3.3 Defect in GalNAc4S-6ST affected expression of type I collagen, decorin and versican

In Fig. 1C, immunostainings of Type I collagen are shown. On 2 days after the last CCl_4_ injection, Type I collagen was found in portal tracts and boundary of hepatic lobules, and was weakly found in sinusoids in wild type mouse liver. In GalNAc4S-6ST KO mouse liver, Type I collagen was detected in essentially the same regions as wild type mouse liver, but sinusoids were stained very weakly. On 3 weeks, the stained areas spread to the periphery of hepatocytes and sinusoids in wild type mouse liver, while most of Type I collagen still remained on portal tracts and boundary of hepatic lobules in GalNAc4S-6ST KO mouse liver. On 6 weeks, Type I collagen was detected only weakly in the portal tracts, boundary of hepatic lobules and sinusoids in both wild type and GalNAc4S-6ST KO mouse liver.

**Fig. 4:**
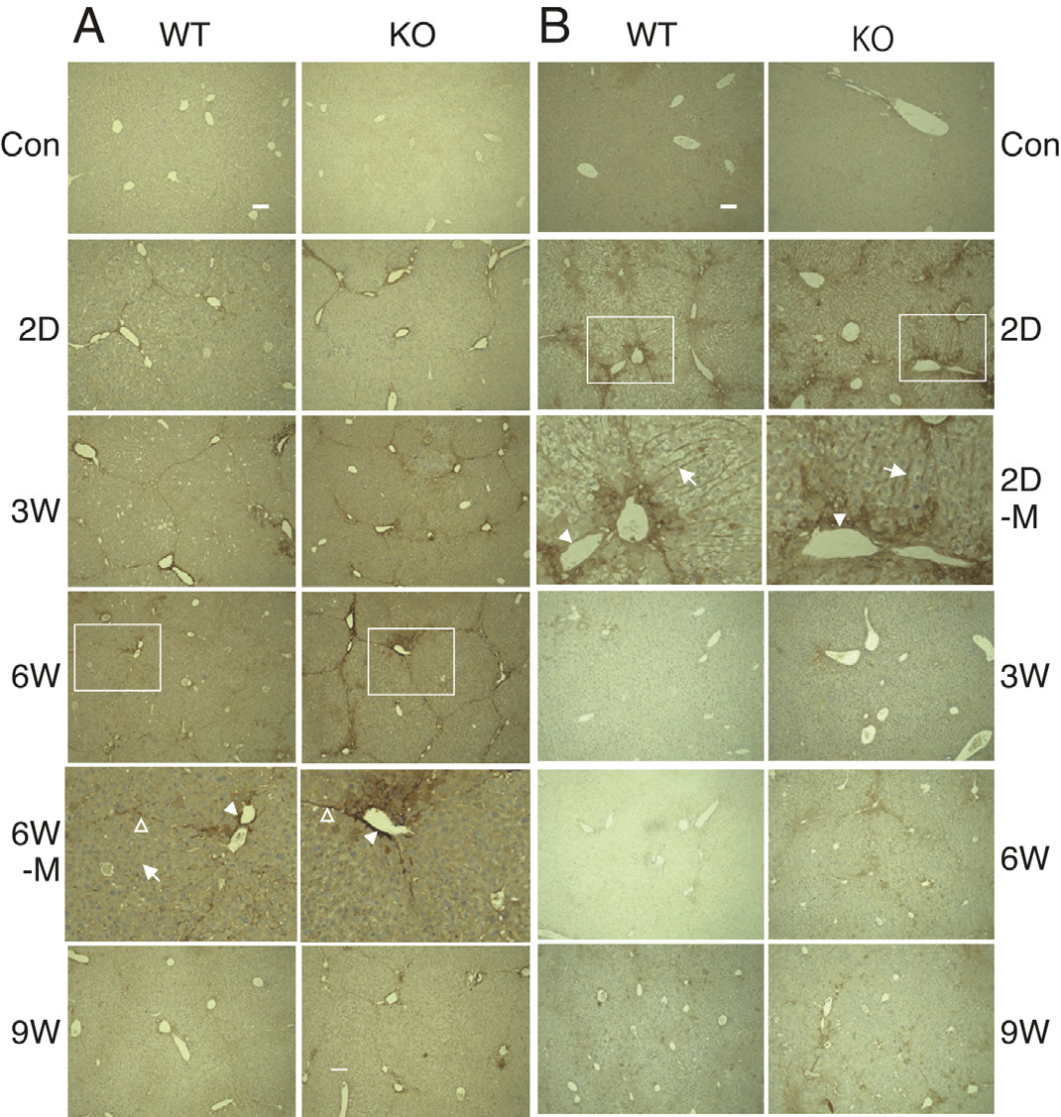
Detection of decorin and versican in the livers obtained from wild type mice (WT) and GalNAc4S-6ST KO mice (KO). Magnified pictures of regions surrounded by rectangles in 6W in (A) and 2D in (B) are shown in 6W-M and 2D-M, respectively. Portal tracts, boundaries of hepatic lobule and sinusoids are indicated by filled arrowheads, open arrowheads and arrows, respectively in 6W-M and 2D-M. The results show representative photographs. (A) Immunostaining of decorin with anti-decorin antibody. (B) Immunostaining of versican with anti-versican. Bar: 100 μm.

To determine if the deficiency in GalNAc4S-6ST may influence the expression of decorin under administration of CCl_4_, we stained the livers by anti-decorin antibody (Fig. 4A). On 2 days after cessation of CCl_4_ injection, decorin was found at the boundary of hepatic lobules and portal tracts but not at the periphery of central veins in the livers of both wild type and GalNAc4S-6ST KO mice. On 6 weeks after the last CCl_4_ injection, sinusoids became positive; the positive areas on the sinusoids in wild type mouse liver were much clearer than those in GalNAc4S-6ST KO mouse liver. In contrast, decorin detected in the boundary of hepatic lobules and portal tracts appeared sharper in GalNAc4S-6ST KO mouse liver than in wild type mouse liver. On 9 weeks after the last CCl_4_ injection, portal tracts in GalNAc4S-6ST KO mouse liver still remained positive, whereas most of the positive areas disappeared in wild type mouse liver.

Versican, another extracellular matrix proteoglycan containing CS/DS, was detected in portal tracts, sinusoids and periphery of central vein of the liver 2 days after the last CCl_4_ injection in both wild type and GalNAc4S-6ST KO mouse livers (Fig. 4B). The expression of versican in GalNAc4S-6ST KO mouse liver appeared to be higher than that in wild type mouse liver. On 3 weeks and thereafter, versican was hardly detected in both wild type and GalNAc4S-6ST KO mouse livers. The observation that versican was detected in sinusoids and periphery of central vein on 2 days after the last CCl_4_ injection contrasted markedly with the observation that decorin was nearly undetectable in these regions on the same day.

### 3.4 Expression of mRNA of versican was quite different from those of decorin and biglycan in fibrosis

**Fig. 5:**
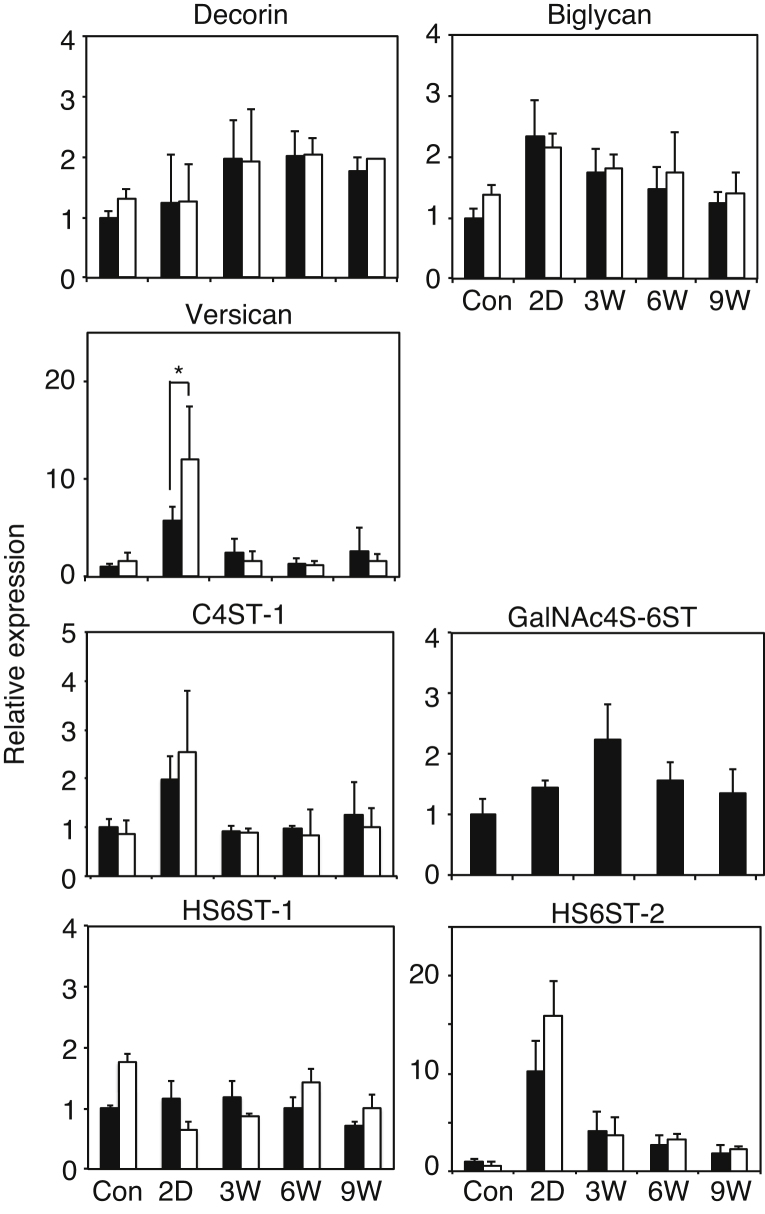
Expression of mRNAs of proteoglycans and sulfotransferases in the livers obtained from wild type mice and GalNAc4S-6ST KO mice. The histograms show the relative expression of the mRNAs of decorin, biglycan, versican (*p < 0.05), C4ST-1, GalNAc4S-6ST, HS6ST-1, and HS6ST-2 in the livers from wild type (filled bar) and KO mice (open bar). Values indicate averages of 3 mice or more, and SDs of data are indicated above each bar.

As shown in Fig. 4, expression of core proteins of decorin and versican was clearly different from each other. To confirm whether such difference may reflect expression patterns of mRNAs of these proteoglycans, we determined expression of mRNAs of decorin and versican (Fig. 5). Expression of biglycan was also determined because liver biglycan was found to bear highly sulfated CS/DS-E [13]. The levels of mRNA of both decorin and biglycan were increased moderately after cessation of CCl_4_ injection; however there were no significant difference between wild type mice and GalNAc4S-6ST KO mice. In contrast, expression of mRNA of versican increased markedly on 2 days after the last CCl_4_ injection and dropped to the control level on 3 weeks; the expression level on 2 days was significantly higher in GalNAc4S-6ST KO mice than in wild type mice. Such expression patterns of mRNA of decorin and versican coincide well with the immunohistochemical detection of these core proteins indicated in Fig. 4.

### 3.5 Expression of mRNAs of sulfotransferases involved in the synthesis of CS/DS-E and HS/heparin in fibrosis

C4ST-1 and GalNAc4S-6ST are involved in the synthesis of CS/DS-E. We determined expression of these sulfotransferases in the liver fibrosis. We also determined expression of HS6ST-1 and HS6ST-2, which are involved in the synthesis of HS/heparin (Fig. 5). The level of mRNA of C4ST-1 and GalNAc4S-6ST showed a peak on 2 days and 3 weeks, respectively, after cessation of CCl_4_ injection. Expression of HS6ST-2 was increased ten times as large as that of control on 2 days, whereas expression of HS6ST-1 hardly changed during fibrosis. No significant differences in the expression of C4ST-1, HS6ST-1 and HS6ST-2 were observed between wild type and GalNAc4S-6ST KO mice.

### 3.6 Change in total amounts and disaccharide composition of CS/DS and HS/heparin on the administration of CCl_4_

**Fig. 6:**
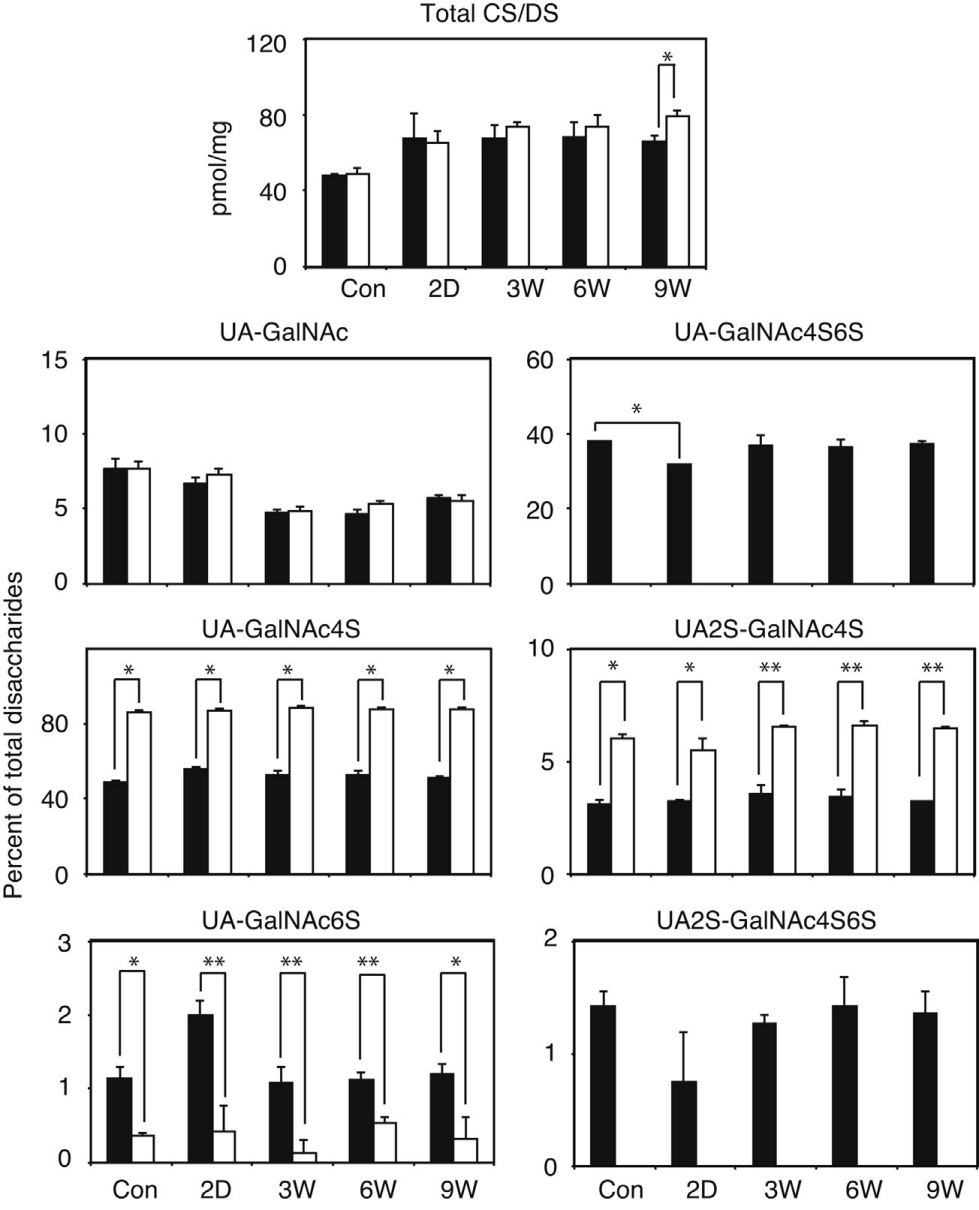
Total amounts and disaccharide compositions of CS/DS obtained from the livers of wild type mice and GalNAc4S-6ST KO mice. The histograms show the total amount (*p < 0.05) of CS/DS, and percentage compositions of unsaturated disaccharide derived from UA-GalNAc, UA-GalNAc4S (*p < 0.0005), UA-GalNAc6S (*p < 0.05, **p < 0.005), UA-GalNAc4S6S (*p < 0.0001), UA2S-GalNAc4S (*p < 0.005, **p < 0.00001), and UA2S-GalNAc4S6S in the CS/DS of wild type (filled bar) and KO mice (open bar). Values indicate averages of 3 mice, and SDs of data are indicated above each bar.

To determine if administration of CCl_4_ may affect amounts and composition of glycosaminoglycans, we measured total amounts and disaccharide composition of CS/DS and HS/heparin obtained from the liver (Fig. 6). After the last CCl_4_ injection, the amounts of CS/DS were significantly increased in both wild type and GalNAc4S-6ST KO mice. A significant difference between wild type and GalNAc4S-6ST KO mice was obtained on 9 weeks. As reported previously, GlcA/IdoA (UA)-GalNAc(4,6-bissulfate) (GalNAc4S6S) unit disappeared completely and instead UA-GalNAc(4-sulfate) (GalNAc4S) unit was increased in GalNAc4S-6ST KO mice. In wild type mice, UA-GalNAc4S6S unit was decreased on 2 days after the last CCl_4_ injection, and recovered to the control level on 3 weeks and thereafter. Differences in the proportions of UA-GalNAc6S and GlcA/IdoA (2-sulfate) (UA2S)-GalNAc4S between wild type mice and GalNAc4S-6ST KO mice were also significant at all time points; however, reasons for these differences remain obscure. The total amount and disaccharide composition of heparan sulfate in the liver varied only slightly on the administration of CCl_4_; nevertheless, proportion of UA2S-GlcNS6S was significantly lower in GalNAc4S-6ST KO mice than in wild type mice (data not shown).

### 3.7 Effects of defect in GalNAc4S-6ST on the expression of mRNAs involved in the synthesis and degradation of collagens

**Fig. 7:**
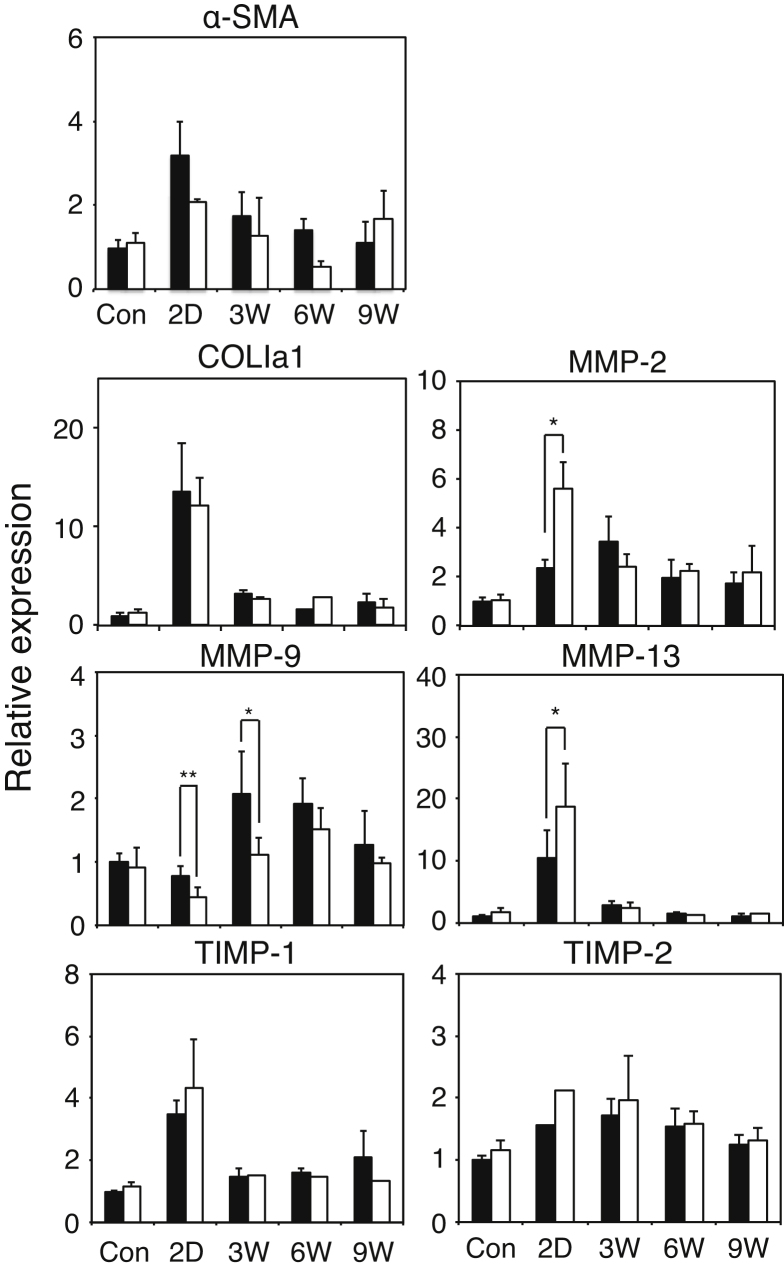
Expression of mRNAs of MMPs and TIMPs in the livers obtained from wild type mice and GalNAc4S-6ST KO mice. The histograms show the relative expression of the mRNAs of COL1a1, MMP-2 (*p < 0.001), MMP-9 (*p < 0.05, **p < 0.01), MMP-13 (*p < 0.01), TIMP-1, TIMP-2 and α-SMA in the livers from wild type (filled bar) and KO mice (open bar). Values indicate averages of 3 mice except for the expression of TIMP-2 in KO mice on 2 days in which one sample was available. SDs of data are indicated above each bar.

As indicated in Fig. 1 and Fig. 2, defect in GalNAc4S-6ST appeared to promote liver fibrosis and to delay recovery from fibrosis in CCl_4_-treated mice. From these observations, it may be rational to postulate that the rate of synthesis and/or degradation of collagen might be affected in GalNAc4S-6ST KO mice. To confirm such possibilities, we determined expression of mRNAs of α-smooth muscle actin (α-SMA), COL1a1, MMPs and TIMPs (Fig. 7). The expression of α-SMA that is thought to reflect activation of HSCs peaked on 2 days after the last CCl_4_ injection, but there were no significant differences in the expression of α-SMA between wild type mice and GalNAc4S-6ST KO mice. The expression of COL1a1 on 2 days after the last CCl_4_ injection was ten times as large as that of control, and decreased precipitously. There were no significant differences in the expression of COL1a1 between wild type mice and GalNAc4S-6ST KO mice at any time points, suggesting that the rate of synthesis of Type I collagen should not be affected by the defect in GalNAc4S-6ST. Expression of MMPs showed species-specific pattern. Expression of MMP-2 and MMP-13 peaked on 2 day after cessation of CCl_4_ injection and decreased thereafter. On 2 days after the last CCl_4_ injection, expression of these MMPs of was significantly higher in GalNAc4S-6ST KO mice than in wild type mice. Expression of MMP-13 returned to the control level on 6 weeks, whereas expression of MMP-2 still showed an elevated level on 9 weeks. Unlike MMP-13 and MMP-2, expression of MMP-9 in wild type mice peaked on 3 weeks after the last CCl_4_ injection, whereas, expression of MMP-9 in GalNAc4S-6ST KO mice did not show clear peak on 3 weeks and was significantly lower than that in wild type mice (Fig. 7). Expression pattern of TIMP-1 was similar to that of MMP-13. Expression of TIMP-2 was increased moderately on 2 days to 3 weeks. No significant differences in the expression of TIMP-1 and TIMP-2 were found between wild type mice and GalNAc4S-6ST KO mice at any time points.

### 3.8 Liver MMP-9 activity was lower in GalNAc4S-6ST KO mice than in wild type mice

**Fig. 8:**
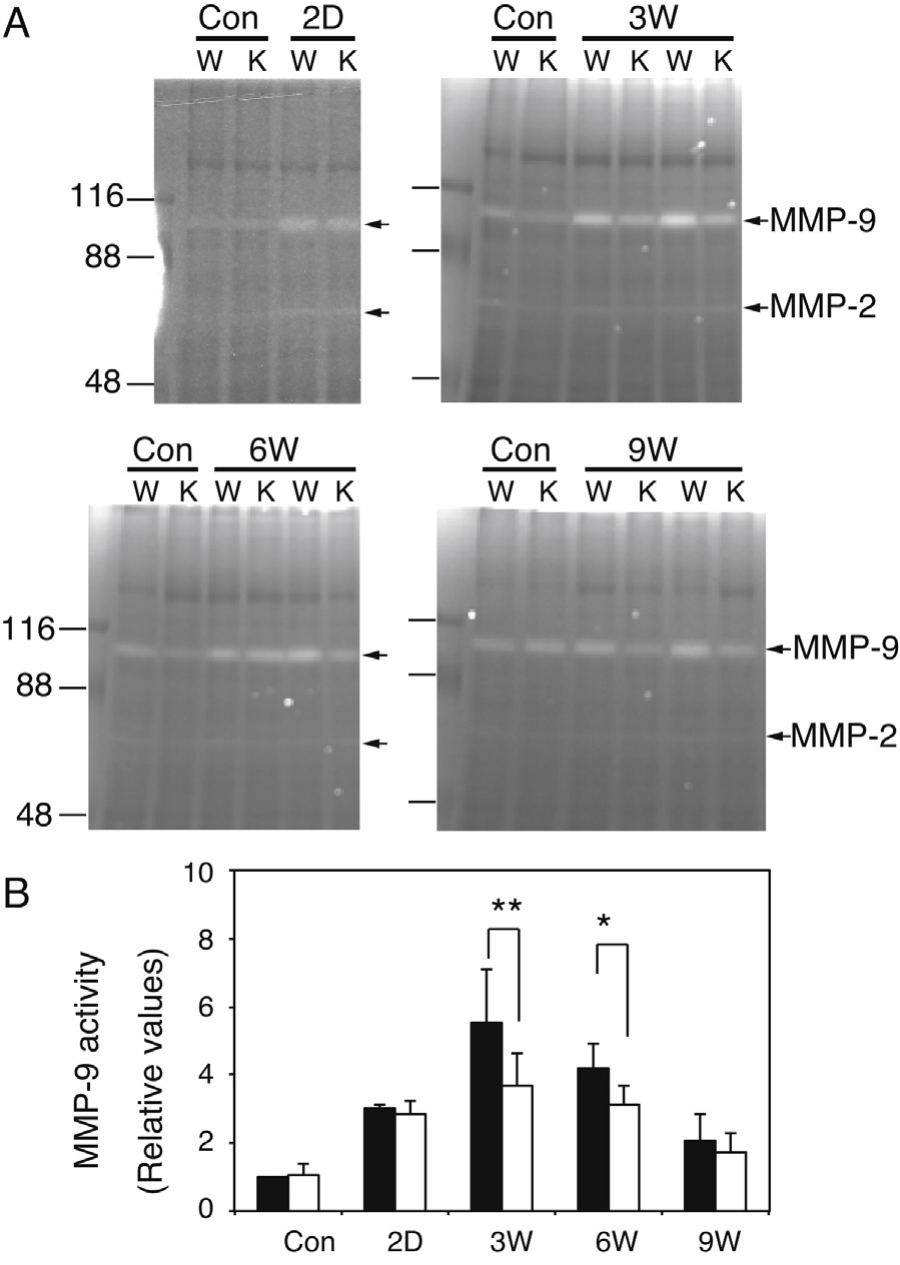
Zymography of liver extracts obtained from wild type mice and GalNAc4S-6ST KO mice. (A) Representative photographs of zymography of wild type (W) and KO (K) mice. Bands corresponding to proMMP-9 and MMP-2 are indicated by arrows. (B) The histograms show the relative values of MMP-9 (*p < 0.05, **p < 0.03) from wild type (filled bar) and KO mice (open bar). Values indicate averages of 3 mice, and SDs of data are indicated above each bar.

As shown in Fig. 7, expression of MMP-9 mRNA was significantly lower in GalNAc4S-6ST KO mice than in wild type mice. To confirm that MMP-9 activity was also decreased in GalNAc4S-6ST KO mice, we determined MMP-9 activity included in the extracts from the livers by using zymography. In Fig. 8A, representative results are shown. A band detected at 105 kDa and a weak band at 68 kDa are thought to correspond to proMMP-9 and MMP-2, respectively. The densities of 105 kDa bands were quantitated by NIH ImageJ64 and expressed in Fig. 8B. The activities of MMP-9 on 3 weeks after the last CCl_4_ injection were more than 3 times higher than those of the controls in both wild type mice and GalNAc4S-6ST KO mice. On 3 weeks and 6 weeks, the activities of MMP-9 were significantly lower in GalNAc4S-6ST KO mice than in wild type mice. These observations appeared to indicate that upregulation of MMP-9 in the CCl_4_-induced liver fibrosis was hampered in GalNAc4S-6ST KO mice.

### 3.9 Defect in GalNAc4S-6ST had little effect on the expression of mRNAs of COL1a1 and MMP-13 in the cultured hepatic stellate cells

**Fig. 9:**
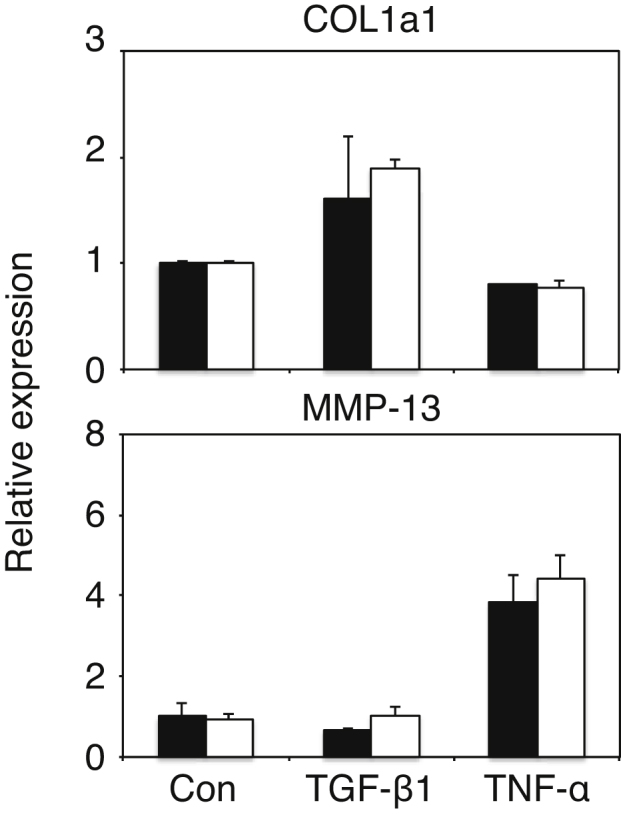
Expression of mRNAs of COL1a1 and MMP-13 in the hepatic stellate cells (HSCs) obtained from the livers of wild type mice and GalNAc4S-6ST KO mice. The histograms show the relative expression of the mRNAs of COL1a1 and MMP-13 of wild type (filled bar) and KO cells (open bar). Values indicate averages of 3 or more than 3 mice, and SDs of data are indicated above each bar.

As shown in Fig. 7, expression of MMP-2 and MMP-13 mRNA showed a peak on 2 days after the last CCl_4_ injection, and expression of these MMPs was significantly higher in GalNAc4S-6ST KO mice than that in wild type mice. On the other hand, expression of α-SMA mRNA also showed a peak on 2 days after the last CCl_4_ injection. α-SMA is known to be a marker of the activated HSC, suggesting that expression of these MMP genes may be due partly to activated HSCs. To examine a possibility that defect in GalNAc4S-6ST may affect the gene expression in HSC, we prepared HSCs from mouse liver and determined expression of mRNAs of COL1a1 and MMP-13 under stimulation with TGF-β1 or TNF-α. As shown in Fig. 9, expression of COL1a1 was increased with TGF-β1 and expression of MMP-13 was increased with TNF-α. However, no significant differences in the expression of these genes in HSCs were observed between wild type mice and GalNAc4S-6ST KO mice. From these observations, it appears to be unlikely that defect in GalNAc4S-6ST affects the gene expression in HSC.

## 4 Discussion

In CCl_4_-induced liver fibrosis, enhanced fibrosis and delayed recovery from fibrosis were observed in GalNAc4S-6ST KO mice (Fig. 1 and Fig. 2). On 3 weeks after the last injection of CCl_4_ and thereafter, expression of mRNA of MMP-9 and MMP-9 activity were both lower in GalNAc4S-6ST KO mice than in wild type mice. On the other hand, in thioacetamide-induced liver fibrosis, liver fibrosis was enhanced and the tissue repair delayed in decorin KO mice, and MMP-9 activity was lower in decorin KO mice [19]. Considering these overlapped observations, it might be possible that CS/DS-E attached to decorin may bear some part of functions of decorin proteoglycan in liver fibrosis. Decorin binds to TGF-β1 and interfere with TGF-β1 activity [26]. Decorin inhibits proliferation of macrophages and protects macrophages from the induction of apoptosis, and the effects of decorin on macrophage activation were mediated through abolishing the binding of TGF-β to macrophages [27]. TGF-β is a heparin-binding protein [28]. Since CS/DS-E is a highly sulfated glycosaminoglycan similar to heparin, it might be possible that CS/DS-E attached to decorin could bind TGF-β and modify the activity of decorin toward TGF-β.

As shown in Fig. 7, expression of MMP-13 and MMP-2 mRNA peaked on 2 days after cessation of CCl_4_ administration and the expression degree of these MMP mRNAs was higher in GalNAc4S-6ST KO mice than in wild type mice. Elevation of MMP-13 and MMP-2 mRNA might reflect the activation of fibrolysis. Alternatively, elevation of these mRNA might be accompanied with acute liver injury because MMP-13 was reported to express in CCl_4_-induced acute liver injury [29], and because expression of MMP-2 was reported to increase in the aggressive phase of liver fibrosis but decrease in the repair stage [30, 31]. From the results of serum ALT activity, the livers on 2 days after cessation of CCl_4_ administration are thought to be in acute phase of liver injury. The activity of serum ALT at 24 h and expression of MMP-13 and MMP-2 mRNA on 2 days after cessation of CCl_4_ administration are both higher in GalNAc4S-6ST KO mice than in wild type mice, suggesting that defect in GalNAc4S-6ST may make the liver more susceptible to the CCl_4_-induced liver injury. The reasons why expression of MMP-2 and MMP-13 was higher in GalNAc4S-6ST KO mice than in wild type mice remain obscure. It is well known that activation of hepatic stellate cells (HSCs) plays important roles in liver fibrosis [32]. Activation of HSC might be different between GalNAc4S-6ST KO mice and wild type mice, and consequently expression of MMP-13 and MMP-2 mRNA on 2 days after cessation of CCl_4_ administration might become different between GalNAc4S-6ST KO mice and wild type mice [33, 34, 35]. However, this possibility seems to be unlikely because there were no significant differences in the expression of COL1a1 and MMP-13 genes under stimulation with TGF-β1 or TNF-α between wild type mice and GalNAc4S-6ST KO mice as shown in Fig. 9. MMP-13 was reported to express in scar-associated macrophages in the liver [36]. It remains to be studied whether expression of MMP-13 in scar-associated macrophages might be affected by the defect of GalNAc4S-6ST.

As shown in Fig. 7 and Fig. 8, expression of MMP-9 showed a peak during the resolution stage and was lower in GalNAc4S-6ST KO mice than in wild type mice. In this report it was not clarified why expression of MMP-9 was lower in GalNAc4S-6ST KO mice than in wild type mice. MMP-9 was shown to express in macrophages [37, 38] and dendritic cells [39], and to contribute to the repair from fibrosis [39]. Because CS/DS-E has been found to express in macrophages derived from monocytes [40], it might be possible that defect in GalNAc4S-6ST results in disappearance of CS/DS-E present in macrophage, and influences expression of MMP-9 in macrophages or dendritic cells through unidentified mechanism.

In wild type mice, UA-GalNAc4S6S unit was decreased on 2 days after the last CCl_4_ injection, and recovered to the control level on 3 weeks and thereafter (Fig. 6). These changes in the proportion of UA-GalNAc4S6S unit in CS/DS may reflect the observation that expression of versican was increased 2 days after the last CCl_4_ injection (Fig. 4B). Augmented expression of versican in the CCl_4_-induced liver fibrosis has been observed [41]. Versican was thought to contain minimal amount of highly sulfated CS/DSs because versican core protein could not be identified in mouse liver proteoglycans containing highly sulfated CS/DS [13]. If CS/DS-E favors the recovery from fibrosis as shown in Fig. 1 and Fig. 2, expression of C4ST-1 and GalNAc4S-6ST that are both involved in the synthesis of CS/DS-E should be increased when liver fibrosis proceeded. The observation that expression of these sulfotransferases actually increased after the administration of CCl_4_ (Fig. 5) seem to support the idea that CS/DS-E may play a certain role in the recovery process. Expression of HS6ST-2 was markedly increased on 2 days but expression of HS6ST-1 was not, suggesting that heparin-containing mast cells may be increased in the acute stage, because HS6ST-2 but not HS6ST-1 was shown to be involved mainly in the synthesis of highly sulfated heparin [42].

Localization of decorin and versican in the liver on 2 days after cessation of CCl_4_ administration was quite different from each other; decorin was rarely present on the periphery of the central vein and sinusoids, but versican was clearly detected in these regions. Expression of mRNA of decorin and versican was also much different from each other; expression of decorin was increased moderately after cessation of CCl_4_ injection, whereas expression of versican was markedly increased 2 days after the last CCl_4_ injection. It is reported that the expression of versican gene was higher in M1 macrophages than that in M2 macrophages [43, 44]. The increased expression of versican on 2 days after the last CCl_4_ injection might reflect the polarization of macrophages.

Complete disappearance of UA-GalNAc4S6S disaccharides in GalNAc4S-6ST KO mice was accompanied with increase in UA-GalNAc4S and UA2S-GalNAc4S disaccharides and decrease in UA-GalNAc6S disaccharides (Fig. 6). It might be possible that changes in disaccharides other than UA-GalNAc4S6S affect liver fibrosis and repair significantly. However, this possibility seems to be unlikely because interaction of various growth factors and cytokines with UA-GalNAc4S was much lower than that with UA-GalNAc4S6S [45] and because both UA2S-GalNAc4S and UA-GalNAc6S are only minor components of liver CS/DS.

In this report, we showed a possibility that disappearance of CS/DS-E resulted from defect in GalNAc4S-6ST may be involved not only in the progression of liver fibrosis but also in the recovery process in CCl_4_-treated mice. CS/DS-E was found in macrophages derived from monocytes [40], and macrophages play important roles not only in inflammation but also in tissue repair and/or regeneration [46]. It remains to be determined whether CS/DS-E takes part in these processes through affecting functions of macrophages. Comparison of differently activated macrophages obtained from wild type and GalNAc4S-6ST KO mice may provide some clues about this issue.

## Declarations

### Author contribution statement

Hiroko Habuchi: Conceived and designed the experiments; Performed the experiments; Analyzed and interpreted the data.

Takahiro Ushida: Analyzed and interpreted the data; Contributed reagents, materials, analysis tools or data.

Osami Habuchi: Conceived and designed the experiments; Performed the experiments; Analyzed and interpreted the data; Contributed reagents, materials, analysis tools or data; Wrote the paper.

### Funding statement

This work was supported by Japanese Government Grant-in-aid for Scientific Research (C) 23570175 (OH).

### Competing interest statement

The authors declare no conflict of interest.

### Additional information

No additional information is available for this paper.

## References

[1] Clement A.M., Sugahara K., Faissner A. (1999). Chondroitin sulfate E promotes neurite outgrowth of rat embryonic day 18 hippocampal neurons. Neurosci. Lett..

[2] Brown J.M., Xia J., Zhuang B., Cho K.S., Rogers C.J., Gama C.I., Rawat M., Tully S.E., Uetani N., Mason D.E., Tremblay E., Habuchi O., Chen D.F., Hsieh-Wilson L.C. (2012). A sulfated carbohydrate epitope inhibits axon regeneration after injury. Proc. Natl. Acad. Sci. USA.

[3] Karumbaiah L., Anand S., Thazhath R., Zhong Y., McKeon R.J., Bellamkonda R.V. (2011). Targeted downregulation of N-acetylgalactosamine 4-sulfate 6-O-sulfotransferase (GalNAc4S6ST) significantly mitigates chondroitin sulfate proteoglycan (CSPG) mediated inhibition. Glia.

[4] Kobayashi T., Yan H., Kurahashi Y., Ito Y., Maeda H., Tada T., Hongo K., Nakayama J. (2013). Role of GalNAc4S-6ST in Astrocytic Tumor Progression. PLoS One.

[5] Basappa, Murugan S., Sugahara K.N., Lee C.M., ten Dam G.B., van Kuppevelt T.H., Miyasaka M., Yamada S., Sugahara K. (2009). Involvement of chondroitin sulfate E in the liver tumor focal formation of murine osteosarcoma cells. Glycobiology.

[6] Iida J., Dorchak J., Clancy R., Slavik J., Ellsworth R., Katagiri Y., Pugacheva E.N., van Kuppevelt T.H., Mural R.J., Cutler M.L., Shriver C.D. (2015). Role for chondroitin sulfate glycosaminoglycan NEDD9-mediated breast cancer cell growth. Exp. Cell Res..

[7] Willis C.M., Klüppel M. (2014). Chondroitin Sulfate-E Is a Negative Regulator of a Pro-Tumorigenic Wnt/Beta-Catenin-Collagen 1 Axis in Breast Cancer Cells. PLoS One.

[8] Miyazaki T., Miyauchi S., Tawada A., Anada T., Matsuzaka S., Suzuki O. (2008). Oversulfated Chondroitin Sulfate-E Binds to BMP-4 and Enhances Osteoblast Differentiation. J. Cell Physiol..

[9] Koike T., Mikami T., Shida M., Habuchi O., Kitagawa H. (2015). Chondroitin sulfate-E mediates estrogen-induced osteoanabolism. Sci. Rep..

[10] Miyazaki T., Miyauchi S., Anada T., Tawada A., Suzuki O. (2015). Chondroitin sulfate-E binds to both osteoactivin and integrin αVβ3 and inhibits osteoclast differentiation. J. Cell Biochem..

[11] Watanabe K., Arumugam S., Sreedhar R., Thandavarayan R.A., Nakamura T., Nakamura M., Harima M., Yoneyama H., Suzuki K. (2015). Small interfering RNA therapy against carbohydrate sulfotransferase 15 inhibits cardiac remodeling in rats with dilated cardiomyopathy. Cell Signal.

[12] Kawamura D., Funakoshi T., Mizumoto S., Sugahara K., Iwasaki N. (2014). Sulfation patterns of exogenous chondroitin sulfate affect chondrogenic differentiation of ATDC5 cells. J. Orthop. Sci..

[13] Ohtake-Niimi S., Kondo S., Ito T., Kakehi S., Ohta T., Habuchi H., Kimata K., Habuchi O. (2010). Mice deficient in N-acetylgalactosamine 4-sulfate 6-o-sulfotransferase are unable to synthesize chondroitin/dermatan sulfate containing N-acetylgalactosamine 4,6-bissulfate residues and exhibit decreased protease activity in bone marrow-derived mast cells. J. Biol. Chem..

[14] Deakin J.A., Lyon M. (2008). A simplified and sensitive fluorescent method for disaccharide analysis of both heparan sulfate and chondroitin/dermatan sulfates from biological samples. Glycobiology.

[15] Koshiishi I., Motoki K., Qiu G., Imanari T. (1993). Structural diversity of mammalian hepatic dermatan sulfates. Biol. Pharm. Bull..

[16] Dudás J., Kovalszky I., Gallai M., Nagy J.O., Schaff Z., Knittel T., Mehde M., Neubauer K., Szalay F., Ramadori G. (2001). Expression of decorin, transforming growth factor-beta 1, tissue inhibitor metalloproteinase 1 and 2, and type IV collagenases in chronic hepatitis. Am. J. Clin. Pathol..

[17] Gallai M., Kovalszky I., Knittel T., Neubauer K., Armbrust T., Ramadori G. (1996). Expression of extracellular matrix proteoglycans perlecan and decorin in carbon-tetrachloride-injured rat liver and in isolated liver cells. Am. J. Pathol..

[18] Meyer D.H., Krull N., Dreher K.L., Gressner A.M. (1992). Biglycan and decorin gene expression in normal and fibrotic rat liver: cellular localization and regulatory factors. Hepatology.

[19] Baghy K., Dezso K., László V., Fullár A., Péterfia B., Paku S., Nagy P., Schaff Z., Iozzo R.V., Kovalszky I. (2011). Ablation of the decorin gene enhances experimental hepatic fibrosis and impairs hepatic healing in mice. Lab. Invest..

[20] Nadanaka S., Kagiyama S., Kitagawa H. (2013). Roles of EXTL2, a member of the EXT family of tumour suppressors, in liver injury and regeneration processes. Biochem. J..

[21] Koshiishi I., Takenouchi M., Imanari T. (1999). Structural Characteristics of Oversulfated　Chondroitin/Dermatan Sulfates in the Fibrous Lesions of the Liver with Cirrhosis. Arch. Biochem. Biophys..

[22] Kivirikko K.I., Laitinen O., Prockop D.J. (1967). Modifications of a specific assay for hydroxyproline in urine. Anal. Biochem..

[23] Nagatani Y., Muto Y., Sato H., Iijima M. (1986). An improved method for the determination of hydroxyproline. Yakugaku Zasshi.

[24] Toyoda H., Kinoshita-Toyoda A., Fox B., Selleck S. (2000). B Structural analysis of glycosaminoglycans in Drosophila and Caenorhabditis elegans and demonstration that tout-velu, a Drosophila gene related to EXT tumor suppressors, affects heparan sulfate in vivo. J. Biol. Chem..

[25] Maschmeyer P., Flach M., Winau F. (2011). Seven steps to stellate cells. J. Vis. Exp..

[26] Yamaguchi Y., Mann D.M., Ruoslahti E. (1990). Negative regulation of transforming growth factor-beta by the proteoglycan decorin. Nature.

[27] Comalada M., Cardó M., Xaus J., Valledor A.F., Lloberas J., Ventura F., Celada A. (2003). Decorin reverses the repressive effect of autocrine-produced TGF-beta on mouse macrophage activation. J. Immunol..

[28] McCaffrey T.A., Falcone D.J., Du B. (1992). Transforming growth factor-beta 1 is a heparin-binding protein: identification of putative heparin-binding regions and isolation of heparins with varying affinity for TGF-beta 1. J. Cell Physiol..

[29] Yata Y., Takahara T., Furui K., Zhang L.P., Watanabe A. (1999). Expression of matrix metalloproteinase-13 and tissue inhibitor of metalloproteinase-1 in acute liver injury. J. Hepatol..

[30] Watanabe T., Niioka M., Ishikawa A., Hozawa S., Arai M., Maruyama K., Okada A., Okazaki I. (2001). Dynamic change of cells expressing MMP-2 mRNA and MT1-MMP mRNA in the recovery from liver fibrosis in the rat. J. Hepatol..

[31] Takahara T., Furui K., Funaki J., Nakayama Y., Itoh H., Miyabayashi C., Sato H., Seiki M., Ooshima A., Watanabe A. (1995). Increased expression of matrix metalloproteinase-II in experimental liver fibrosis in rats. Hepatology.

[32] Friedman S.L. (2008). Hepatic stellate cells: protean, multifunctional, and enigmatic cells of the liver. Physiol. Rev..

[33] Arthur M.J., Friedman S.L., Roll F.J., Bissell D.M. (1989). Lipocytes from normal rat liver release a neutral metalloproteinase that degrades basement membrane (type IV) collagen. J. Clin. Invest..

[34] Arthur M.J., Stanley A., Iredale J.P., Rafferty J.A., Hembry R.M., Friedman S.L. (1992). Secretion of 72 kDa type IV collagenase/gelatinase by cultured human lipocytes. Analysis of gene expression, protein synthesis and proteinase activity. Biochem. J..

[35] Iredale J.P., Benyon R.C., Arthur M.J., Ferris W.F., Alcolado R., Winwood P.J., Clark N., Murphy G. (1996). Tissue inhibitor of metalloproteinase-1 messenger RNA expression is enhanced relative to interstitial collagenase messenger RNA in experimental liver injury and fibrosis. Hepatology.

[36] Fallowfield J.A., Mizuno M., Kendall T.J., Constandinou C.M., Benyon R.C., Duffield J.S., Iredale J.P. (2007). Scar-Associated Macrophages Are a Major Source of Hepatic Matrix Metalloproteinase-13 and Facilitate the Resolution of Murine Hepatic Fibrosis. J. Immunol..

[37] Winwood P.J., Schuppan D., Iredale J.P., Kawser C.A., Docherty A.J., Arthur M.J. (1995). Kupffer cell-derived 95-kd type IV collagenase/gelatinase B: characterization and expression in cultured cells. Hepatology.

[38] Knittel T., Mehde M., Kobold D., Saile B., Dinter C., Ramadori G. (1999). Expression patterns of matrix metalloproteinases and their inhibitors in parenchymal and non-parenchymal cells of rat liver: regulation by TNF-alpha and TGF-beta1. J. Hepatol..

[39] Jiao J., Sastre D., Fiel M.I., Lee U.E., Ghiassi-Nejad Z., Ginhoux F., Vivier E., Friedman S.L., Merad M., Aloman C. (2012). Dendritic cell regulation of carbon tetrachloride-induced murine liver fibrosis regression. Hepatology.

[40] Uhlin-Hansen L., Eskeland T., Kolset S.O. (1989). Modulation of the expression of chondroitin sulfate proteoglycan in stimulated human monocytes. J. Biol. Chem..

[41] Bukong T.N., Maurice S.B., Chahal B., Schaeffer D.F., Winwood P.J. (2016). Versican: a novel modulator of hepatic fibrosis. Lab. Invest..

[42] Anower-E-Khuda M.F., Habuchi H., Nagai N., Habuchi O., Yokochi T., Kimata K. (2013). Heparan sulfate 6-O-sulfotransferase isoform-dependent regulatory effects of heparin on the activities of various proteases in mast cells and the biosynthesis of 6-O-sulfated heparin. J. Biol. Chem..

[43] Martinez F.O., Gordon S., Locati M., Mantovani A. (2006). Transcriptional profiling of the human monocyte-to-macrophage differentiation and polarization: new molecules and patterns of gene expression. J. Immunol..

[44] Chang M.Y., Tanino Y., Vidova V., Kinsella M.G., Chan C.K., Johnson P.Y., Wight T.N., Frevert C.W. (2014). A rapid increase in macrophage-derived versican and hyaluronan in infectious lung disease. Matrix Biol..

[45] Mizumoto S., Fongmoon D., Sugahara K. (2013). Interaction of chondroitin sulfate and dermatan sulfate from various biological sources with heparin-binding growth factors and cytokines. Glycoconj J..

[46] Chazaud B. (2014). Macrophages: Supportive cells for tissue repair and regeneration. Immunobiology.

